# Impact of examined lymph node count on survival outcomes in patients with stage T1-2N0M0 small cell lung cancer undergoing surgery: A retrospective cohort study

**DOI:** 10.1097/MD.0000000000038381

**Published:** 2024-05-31

**Authors:** Xin-Biao Wan, Huan-Wei Liang, Yang Liu, Wei Huang, Xin-Bin Pan

**Affiliations:** a Department of Radiation Oncology, Guangxi Medical University Cancer Hospital, Nanning, Guangxi, China.

**Keywords:** examined lymph node, small cell lung cancer, surgery, T1-2N0M0

## Abstract

To explore the relationship between the count of examined lymph nodes (ELNs) and survival outcomes in patients with stage T1-2N0M0 small cell lung cancer (SCLC) after surgical treatment. We analyzed data from patients with SCLC in the Surveillance, Epidemiology, and End Results database. The study focused on examining the correlation between the ELN count and both cancer-specific survival (CSS) and overall survival (OS). This relationship was investigated using restricted cubic spline curves within the framework of multivariable Cox regression models. The cutoff value for both CSS and OS was 7 ELN counts. Patients with ELN < 7 had a median CSS of 64 months, significantly lower than 123 months of patients with ELN ≥ 7 (*P* = .012). Multivariable Cox regression analysis indicated that ELN ≥ 7 was an independent prognostic factor for CSS (hazard ratio = 0.50, 95% confidence interval: 0.30–0.83; *P* = .007). Similarly, Patients with ELN < 7 had a median OS of 41 months for patients with ELN < 7, compared to 103 months for those with ELN ≥ 7 (*P* = .004). Multivariable Cox regression analysis confirmed that ELN ≥ 7 was an independent prognostic factor for OS (hazard ratio = 0.54, 95% confidence interval: 0.36–0.81; *P* = .003). ELN ≥ 7 is recommended as the threshold for evaluating the quality of postoperative lymph node examination and for prognostic stratification in patients with stage T1-2N0M0 SCLC undergoing surgery.

## 1. Introduction

The evolution of lung cancer screening techniques has significantly impacted the detection of early stage small cell lung cancer (SCLC), culminating in a higher frequency of diagnoses at a stage where the disease might be more amenable to treatment.^[1]^ Traditionally, stage T1-2N0M0 SCLC has been predominantly managed with concurrent chemoradiotherapy, following established treatment guidelines.^[2,3]^ Nonetheless, in a substantial subset of cases, the lack of a preoperative biopsy has necessitated a shift in treatment approach towards surgical resection.^[2–5]^ Comparative investigations have highlighted the superior survival outcomes of surgical intervention compared to concurrent chemoradiotherapy, prompting a reconsideration of treatment strategies for this patient group.^[2,3,5]^

Despite these advancements, the postoperative prognosis for SCLC patients remains daunting. Approximately 50% of the individuals undergoing surgical resection experience local-regional recurrences or distant metastases within the initial 5 years following the surgery, emphasizing the aggressive nature of SCLC and the imperative for meticulous postoperative management.^[6–8]^ In this landscape, the precise identification and quantification of prognostic indicators are crucial for customizing patient-centered treatment plans and enhancing survival rates.

In lung cancer therapy, the status of lymph node involvement is a pivotal prognostic element, particularly in resectable cases.^[9]^ The extent of lymph node dissection and accurate nodal staging are essential in formulating an optimal treatment regimen. The International Association for the Study of Lung Cancer, alongside the European Society of Thoracic Surgery, advocates for the examination of at least 6 lymph nodes for thorough assessment in non-small cell lung cancer (NSCLC) cases.^[10,11]^ In contrast, the Chinese Journal of Oncology recommends examining a minimum of 12 examined lymph nodes (ELNs) for adequate staging.^[12]^

However, the specific influence of ELN count on survival outcomes in SCLC remains relatively uncharted, largely due to the uncommon nature of this cancer subtype and the complexities associated with amassing extensive, detailed data.^[13–16]^ Our study endeavors to fill this gap in knowledge by meticulously examining the impact of ELN count on the survival of patients with stage T1-2N0M0 SCLC undergoing surgical treatment. This research not only seeks to elucidate the prognostic value of ELN count in SCLC but also aims to inform surgical and adjuvant treatment modalities for this patient demographic.

## 2. Materials and methods

### 2.1. Data sources

The Surveillance, Epidemiology, and End Results (SEER) program, managed by the National Cancer Institute, provides a comprehensive database chronicling essential data on cancer incidence, mortality, and prevalence. For our retrospective study, we utilized SEER*Stat software (version 8.4.2), accessible at www.seer.cancer.gov/seerstat, to collate detailed information on SCLC patients spanning 2000 to 2020, offering a substantial temporal framework for our analysis.

Ethics approval was waived by the ethics committee/Institutional Review Board of Guangxi Medical University Cancer Hospital.

### 2.2. Study population

Our research concentrated on individuals with histopathologically confirmed SCLC, specifically those classified as stage pT1-2N0M0 after surgical intervention. To ensure specificity in our results, we excluded patients with prior multiple primary cancers. We meticulously collected various clinical characteristics, including demographics (age, sex, race), tumor specifics (primary site, location, grade, T stage), treatment modalities, and ELN count.

### 2.3. Endpoints

The primary endpoint of our investigation was overall survival (OS), defined as the duration from initial diagnosis to death from any cause, as recorded in the SEER database. The secondary endpoint, cancer-specific survival (CSS), entailed the time from diagnosis to death directly attributable to SCLC.

### 2.4. Stratification of examined lymph node count

To ascertain optimal cutoff values for ELN count, we employed restricted cubic spline regression models, facilitating an exploration of the relationships between diverse ELN counts and key survival indicators (CSS and OS) on a continuum. We evaluated these correlations using multivariable Cox regression models, meticulously adjusted for a comprehensive array of confounders, encompassing patient demographics, tumor characteristics, and treatment specifics.

### 2.5. Statistical analysis

We compared clinical factors across different ELN count groups employing the χ^2^ test or Fisher exact test. Kaplan–Meier methods with log-rank tests were utilized to contrast CSS and OS among various ELN count groups. Multivariable Cox regression analysis was executed to pinpoint independent prognostic factors, adjusting for age, sex, race, primary site, tumor location, tumor grade, T stage, treatments, and ELN count groups.

To mitigate selection bias in comparing CSS and OS across ELN count groups, we conducted a matched case–control analysis using propensity score matching (PSM). This involved one-to-one matching without replacement within a logistic regression framework, employing the nearest-neighbor matching algorithm with a caliper of 0.1.

Statistical analyses were conducted using SPSS Statistics Version 26.0 (IBM Co., Armonk, NY, USA) and R software (version 4.2.2). A 2-tailed *P* value threshold of <.05 was set for determining statistical significance.

## 3. Results

### 3.1. Patient selection

**Figure 1.**
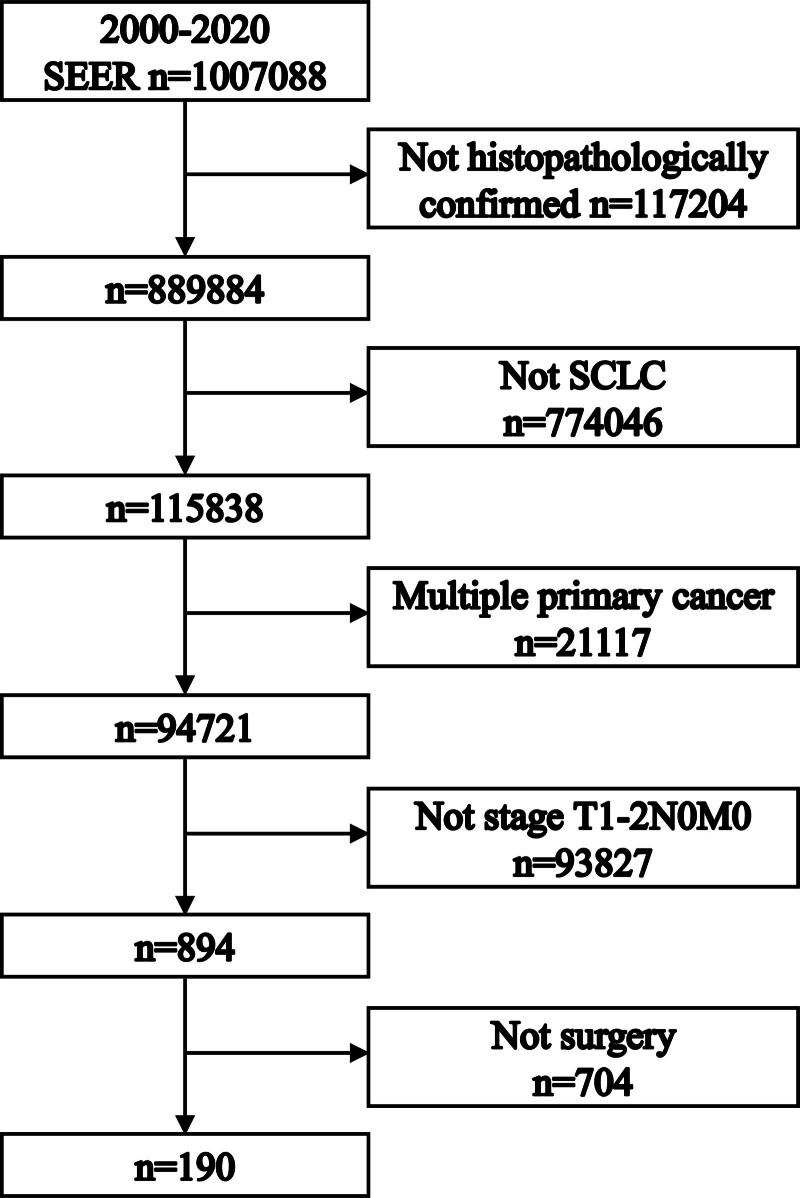
This flowchart details the patient selection process, outlining the criteria and steps followed to identify the study population from the Surveillance, Epidemiology, and End Results (SEER) database, focusing on patients with stage T1-2N0M0 small cell lung cancer (SCLC).

From the SEER database, a total of 1,007,088 lung cancer patients were initially identified. After applying specific selection criteria, 190 patients with stage T1-2N0M0 SCLC were included in our study. The process of patient selection and the applied criteria is illustrated in Figure 1.

### 3.2. Association between ELN count and survival outcomes

**Figure 2.**
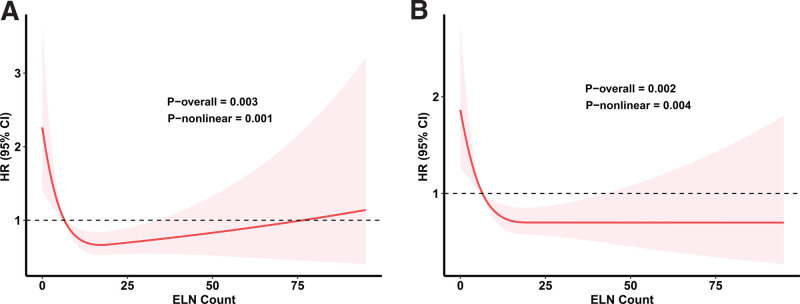
Demonstrates the relationship between examined lymph node (ELN) count and survival outcomes, modeled using restricted cubic spline regression. Panel (A) illustrates the association with cancer-specific survival (CSS), while Panel (B) depicts the association with overall survival (OS).

Our analysis utilized restricted cubic spline regression models, adjusted for potential confounders. This analysis revealed distinct patterns in the association between the ELN count and survival outcomes. For CSS, a U-shaped relationship emerged, indicating that both lower and higher ELN counts were associated with decreased CSS (Fig. 2A). In contrast, for OS, a L-shaped curve was observed, suggesting that higher ELN counts correlated with improved OS (Fig. 2B). The optimal cutoff value for both CSS and OS was identified as 7 ELN counts.

### 3.3. Clinical characteristics and propensity score matching

Baseline clinical characteristics, including age, sex, race, primary site, tumor location, tumor grade, T stage, and treatment modalities, are summarized in Table 1. Notable differences in tumor grade and treatment modalities were observed between patients with ELN counts < 7 (ELN < 7) and those with 7 or more (ELN ≥ 7). Following the application of PSM, these confounders were well balanced between the 2 groups, as shown in the Table 1.

**Table 1:** Patient characteristics.

	The unmatched cohort		*P*	The PSM cohort		*P*
	ELN < 7 (n = 95)	ELN ≥ 7 (n = 95)		ELN < 7 (n = 74)	ELN ≥ 7 (n = 74)	
Age						
<67	44 (46.3%)	50 (52.6%)	.468	40 (54.1%)	39 (52.7%)	.999
≥67	51 (53.7%)	45 (47.4%)		34 (45.9%)	35 (47.3%)	
Sex						
Female	52 (54.7%)	58 (61.1%)	.463	41 (55.4%)	43 (58.1%)	.868
Male	43 (45.3%)	37 (38.9%)		33 (44.6%)	31 (41.9%)	
Race						
White	90 (94.7%)	91 (95.8%)	.999	69 (93.2%)	71 (95.9%)	.759
Black	3 (3.2%)	2 (2.1%)		3 (4.1%)	2 (2.7%)	
others	2 (2.1%)	2 (2.1%)		2 (2.7%)	1 (1.4%)	
Site						
Upper lobe	64 (67.4%)	56 (58.9%)	.068	48 (64.9%)	50 (67.6%)	.670
Middle lobe	9 (9.4%)	3 (3.2%)		5 (6.7%)	2 (2.7%)	
Lower lobe	20 (21.1%)	33 (34.7%)		19 (25.7%)	21 (28.3%)	
Others	2 (2.1%)	3 (3.2%)		2 (2.7%)	1 (1.4%)	
Laterality						
Left	43 (45.3%)	44 (46.3%)	.999	33 (44.6%)	35 (47.3%)	.869
Right	52 (54.7%)	51 (53.7%)		41 (55.4%)	39 (52.7%)	
Grade						
III/IV	67 (70.5%)	52 (54.7%)	.036	47 (63.5%)	47 (63.5%)	.999
I/II/unknown	28 (29.5%)	43 (45.3%)		27 (36.5%)	27 (36.5%)	
T stage						
T1N0M0	66 (69.5%)	57 (60.0%)	.224	48 (64.9%)	48 (64.9%)	.998
T2N0M0	29 (30.5%)	38 (40.0%)		26 (35.1%)	26 (35.1%)	
Treatment						
Surgery alone	37 (38.9%)	28 (29.5%)	.003	31 (41.9%)	23 (31.1%)	.086
Surgery + chemotherapy	24 (25.3%)	46 (48.4%)		21 (28.4%)	34 (45.9%)	
Surgery + radiotherapy	3 (3.2%)	0 (0.0%)		0 (0.0%)	0 (0.0%)	
Surgery + chemoradiotherapy	31 (32.6%)	21 (22.1%)		22 (29.7%)	17 (23.0%)	

ELN = examined lymph node, PSM = propensity score matching.

The median follow-up times were 41 months (interquartile range [IQR]: 16–74 months) for the ELN < 7 group and 64 months (IQR: 33–86 months) for the ELN ≥ 7 group.

### 3.4. Cancer-specific survival

**Figure 3.**
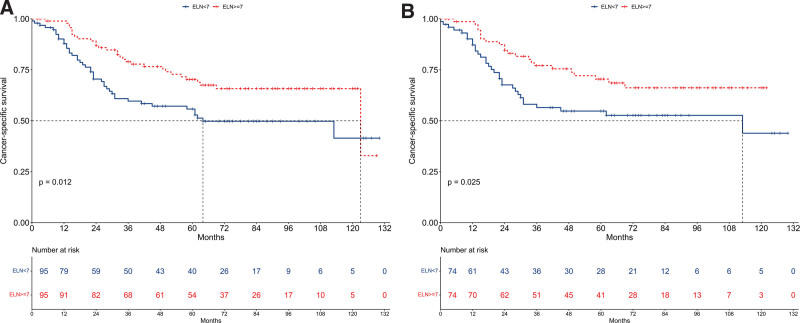
Kaplan–Meier survival curves depicting cancer-specific survival (CSS) comparisons between patients with fewer than 7 examined lymph nodes (ELN < 7) and those with 7 or more examined lymph nodes (ELN ≥ 7). Panel (A) represents data from the unmatched cohort, and Panel (B) shows data from the cohort after propensity score matching (PSM).

**Figure 4.**
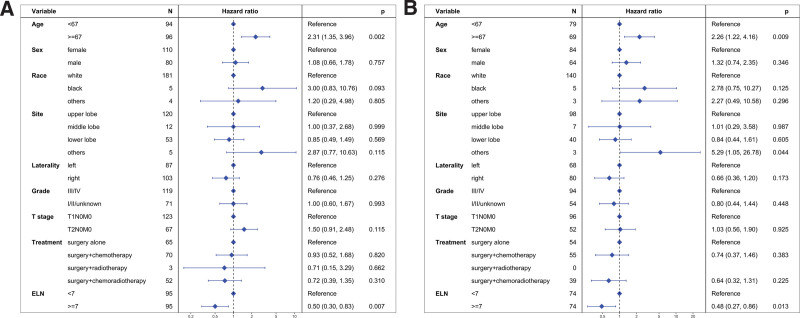
Multivariate regression analysis evaluating various prognostic factors for cancer-specific survival (CSS). Panel (A) includes data from the unmatched cohort, while Panel (B) includes data from the propensity score-matched cohort.

Prior to PSM, the median CSS was 64 months for the ELN < 7 group and 123 months for the ELN ≥ 7 group (Fig. 3A). The ELN ≥ 7 group exhibited a significantly better CSS compared to the ELN < 7 group (hazard ratio [HR] = 0.55, 95% confidence interval [CI]: 0.35–0.88; *P* = .012). Multivariable Cox regression analysis further confirmed that an ELN count of ≥7 was an independent prognostic factor for CSS (HR = 0.50, 95% CI: 0.30–0.83; *P* = .007, Figure 4A).

After PSM, the median CSS was 113 months for the ELN < 7 group and not reached for the ELN ≥ 7 group (Fig. 3B). The ELN ≥ 7 group showed better CSS compared to the ELN < 7 group (HR = 0.54, 95% CI: 0.31–0.93; *P* = .025). Multivariable Cox regression analysis confirmed the ELN ≥ 7 group as an independent prognostic factor for CSS (HR = 0.48, 95% CI: 0.27–0.86; *P* = .013, Fig. 4B).

### 3.5. Overall survival

**Figure 5.**
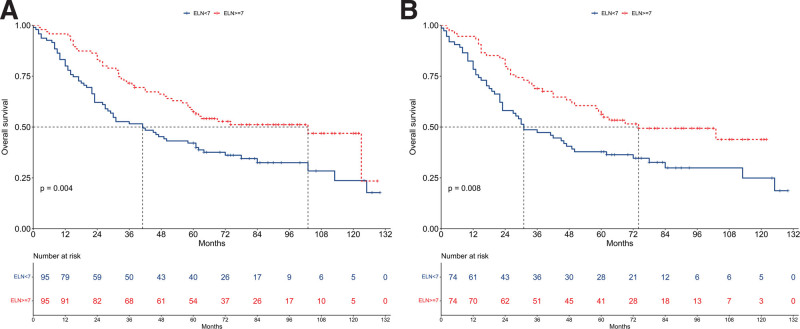
Kaplan–Meier survival curves depicting overall survival (OS) comparisons between patients with fewer than 7 examined lymph nodes (ELN < 7) and those with 7 or more examined lymph nodes (ELN ≥ 7). Panel (A) represents data from the unmatched cohort, and Panel (B) shows data from the cohort after propensity score matching (PSM).

**Figure 6.**
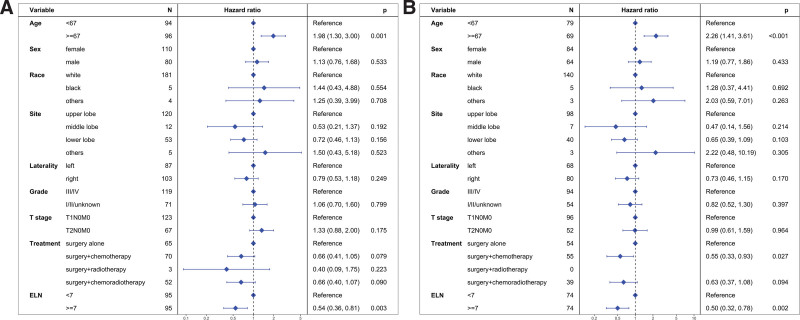
Multivariate regression analysis assessing prognostic factors for overall survival (OS). Panel (A) includes findings from the unmatched cohort, and Panel (B) from the cohort post-propensity score matching.

Before PSM, the median OS was 41 months for the ELN < 7 group and 103 months for the ELN ≥ 7 group (Fig. 5A). The ELN ≥ 7 group demonstrated better OS compared to the ELN < 7 group (HR = 0.58, 95% CI: 0.39–0.84; *P* = .004). Multivariable Cox regression analysis further substantiated that ELN count ≥ 7 was an independent prognostic factor for OS (HR = 0.54, 95% CI: 0.36–0.81; *P* = .003, Fig. 6A).

After PSM, the median OS was 31 months for the ELN < 7 group and 74 months for the ELN ≥ 7 group (Fig. 5B). The ELN ≥ 7 group continued to show a better OS compared to the ELN < 7 group (HR = 0.57, 95% CI: 0.37–0.86; *P* = .008). Multivariable Cox regression analysis reaffirmed that an ELN count ≥ 7 was an independent prognostic factor for OS (HR = 0.50, 95% CI: 0.32–0.78; *P* = .002, Figure 6B).

## 4. Discussion

Our study, utilizing the SEER database, sought to determine the impact of ELN counts on the survival of patients with stage pT1-2N0M0 SCLC undergoing surgical intervention. A pivotal finding is the identification of 7 as the optimal ELN count for both CSS and OS, as determined by sophisticated restricted cubic spline regression models. This finding sets a vital standard in the surgical treatment of early stage SCLC, an area previously devoid of definitive guidelines.

The correlation observed between higher ELN counts and enhanced survival outcomes potentially elucidated a key aspect of lung cancer staging-the phenomenon of stage migration. In cases initially classified as stage N0 disease but with limited lymph node examination, there is a substantial risk of undetected progression to stage N1, N2, or N3 diseases. Consequently, a greater ELN count may prevent under-staging, leading to more precise prognoses and treatment decisions. This indicates that the thoroughness of lymph node examination is as critical as the initial tumor staging for patient outcomes.

It is important to note that while NSCLC and SCLC share a common organ of origin, their clinical and biological behaviors are markedly different. NSCLC, which accounts for approximately 85% of all lung cancer cases,^[17]^ generally has a slower progression and a better prognosis compared to SCLC. These differences are critical when considering surgical interventions and lymph node examination, which are the focal points of our study.

In NSCLC, the influence of ELN on survival outcomes has been more thoroughly explored. It is well-established that adequate nodal dissection and examination are critical for accurate staging and prognostication in NSCLC.^[18]^ Studies have demonstrated that a higher number of examined lymph nodes in NSCLC is associated with improved survival outcomes, likely due to more precise staging and the resultant tailored treatment strategies.^[19–26]^ However, the application of these findings to SCLC is not straightforward due to the more aggressive nature and different treatment paradigms of SCLC.

Furthermore, the surgical approach to NSCLC has evolved significantly over the years, with minimally invasive techniques becoming more prevalent. This evolution in surgical practice may have implications for lymph node examination and retrieval. In contrast, the surgical approach in SCLC remains more conservative due to its aggressive behavior and the greater emphasis on systemic therapy. The difference of surgical approach between NSCLC and SCLC might have impact on the lymph node examination.

Our study sheds light on the prognostic significance of lymph node examination in early stage SCLC, a topic less explored compared to NSCLC. We observed trends similar to NSCLC, where a higher number of examined lymph nodes was associated with better survival outcomes. This finding suggests that, despite the differences in biology and usual treatment courses between SCLC and NSCLC, the extent of lymph node examination might still play a crucial role in both SCLC and NSCLC.

However, it is essential to note that our study indicated the cutoff value of 7 ELN counts for SCLC. In NSCLC, the cutoff value of 16 ELN counts was recommended.^[18]^ The difference of ELN counts between SCLC and NSCLC may be attributed to the aggressive behavior of SCLC and the small sample size of our study. Therefore, our findings might indicate a need to reevaluate the role of surgery and comprehensive lymph node examination in the management of early stage SCLC.

Our study presents several limitations that should be considered. First, the small sample size, primarily due to the infrequency of surgically treatable SCLC, might affect the generalizability of our findings. This emphasizes the need for larger-scale, multi-institutional studies that could both confirm and extend our findings, enhancing the robustness and statistical significance of the data.

Second, it is crucial to acknowledge the inherent constraints associated with the use of data from the SEER database. While the SEER database is a valuable resource for population-based cancer research, providing extensive data on cancer incidence, mortality, and prevalence, it lacks specific details on the criteria for lymph node examination and the nuances of surgical intervention.

The absence of detailed information on the surgical techniques employed, the extent of lymph node dissection, and the criteria used by different surgeons or institutions to determine the number of lymph nodes to be examined, introduces an element of selection bias. This limitation is significant as it potentially affects the generalizability and applicability of our findings. The variability in surgical practices and lymph node examination across different centers might lead to inconsistencies in staging accuracy and could impact survival outcomes.

Third, PSM was effective in balancing measurable confounders such as age, sex, race, primary site, tumor location, tumor grade, T stage, and treatments in our study. However, we recognize that unmeasured variables like smoking status, comorbidities, performance status, precise details of adjuvant therapies, and number of dissected mediastinal lymph node stations were not included in our matching process. These unmeasured variables could have an impact on CSS and OS of the patients with stage T1-2N0M0 SCLC.

The potential influence of these unmeasured confounders on the conclusions drawn from our study is a critical aspect to consider. It suggests that while our findings provide valuable insights into the relationship between ELN count and survival outcomes in stage T1-2N0M0 SCLC, the results should be interpreted with caution. Our results indicate associations rather than causality and serve as a basis for future research rather than definitive evidence.

Given these constraints, our findings, while informative, necessitate a careful and nuanced interpretation. These limitations highlight the need for more comprehensive data collection in cancer registries and highlight the need for multi-institutional studies that can provide more detailed insights into surgical practices and their impact on outcomes in SCLC. Future research should focus on including detailed surgical data and a broader range of clinical variables to enhance understanding of the role of lymph node examination in the management of early stage SCLC.

In conclusion, our research underscores the importance of thorough lymph node examination in SCLC, proposing 7 ELNs as a crucial threshold for accurate N0 staging and prognostic stratification. This benchmark may prompt a reevaluation of current surgical approaches to early stage SCLC and guide future therapeutic strategies toward more personalized and outcome-focused interventions.

## Author contributions

**Conceptualization:** Xin-Biao Wan.

**Writing – original draft:** Xin-Biao Wan.

**Formal analysis:** Huan-Wei Liang.

**Methodology:** Huan-Wei Liang, Yang Liu.

**Resources:** Yang Liu.

**Validation:** Wei Huang.

**Writing – review & editing:** Xin-Bin Pan.
